# The Role of Electron Microscopy in Studying the Continuum of Changes in Membranous Structures during Poliovirus Infection

**DOI:** 10.3390/v7102874

**Published:** 2015-10-12

**Authors:** Evan D. Rossignol, Jie E. Yang, Esther Bullitt

**Affiliations:** Department of Physiology & Biophysics, Boston University School of Medicine, 700 Albany Street, W302, Boston, MA 02118-2526, USA; edr5@bu.edu (E.D.R.); jieay@bu.edu (J.E.Y.)

**Keywords:** poliovirus, RNA replication, RNA virus, membrane, membrane remodeling, electron microscopy, cell morphology, replication organelle

## Abstract

Replication of the poliovirus genome is localized to cytoplasmic replication factories that are fashioned out of a mixture of viral proteins, scavenged cellular components, and new components that are synthesized within the cell due to viral manipulation/up-regulation of protein and phospholipid synthesis. These membranous replication factories are quite complex, and include markers from multiple cytoplasmic cellular organelles. This review focuses on the role of electron microscopy in advancing our understanding of poliovirus RNA replication factories. Structural data from the literature provide the basis for interpreting a wide range of biochemical studies that have been published on virus-induced lipid biosynthesis. In combination, structural and biochemical experiments elucidate the dramatic membrane remodeling that is a hallmark of poliovirus infection. Temporal and spatial membrane modifications throughout the infection cycle are discussed. Early electron microscopy studies of morphological changes following viral infection are re-considered in light of more recent data on viral manipulation of lipid and protein biosynthesis. These data suggest the existence of distinct subcellular vesicle populations, each of which serves specialized roles in poliovirus replication processes.

## 1. Introduction

The focus of this review is on changes in cellular morphology during replication of poliovirus, a positive sense (+) RNA virus of approximately 7500 nucleotides. The poliovirus genome is encapsidated in a non-enveloped icosahedral virion, 28–30 nm in diameter [1]. The virion first attaches to human cells via the receptor CD155 [2], and binding initiates an irreversible conformational change in the capsid [3,4]. This structural change exposes capsid proteins that attach the particle to the membrane in a now receptor-independent manner [5,6], and cell entry occurs via endocytosis that does not require clathrin, but is actin- and tyrosine kinase- dependent [7]. A recent review by Paul and Wimmer [8] includes details on the process of poliovirus replication after cell entry.

Poliovirus RNA replication is always membrane-associated [9] and it has been shown that pre-existing cell membranes do not support genome replication [10]. Therefore, it is not surprising that lipid synthesis is upregulated [11] in infected cells, resulting in dramatic cellular changes. Newly formed membranous structures include specialized sites of RNA replication that have been described as “replication factories” [12]. To facilitate the replication process, replication factories are comprised of cellular membranes that are remodeled and expanded with newly synthesized lipids.

Excellent reviews on RNA viruses have been written recently [13,14,15], including a table in the Neuman *et al.*, review enumerating membrane rearrangements from many (+) RNA viruses [15], as well as in this issue [16]. Our review focuses on the major role that electron microscopy has played in elucidating the extensive protein-lipid structures that are induced to support replication of the picornavirus poliovirus (PV) RNA. In addition, discussion is included on upregulation and changes in the lipid species that are incorporated into replication factories.

Microscopy of poliovirus-infected cells began almost 60 years ago to document the wide-ranging morphological changes that occur within infected cells. Reports beginning in 1956 detailed the first visible cellular changes, appearing by three hours post infection (hpi), using phase light microscopy at 100× magnification [17]. In 1958, electron microscopy was first used to visualize details of cytopathic effects and ultrastructure changes in poliovirus-infected cells [18]. Early electron microscopy studies include morphological data that has not been discussed in detail in recent literature. These early results, and more recent work, are described below, and included in a discussion of the membrane remodeling and expansion required for replication of the poliovirus genome.

## 2. Poliovirus-Modulated Lipid Metabolism and Trafficking, and Their Effect on Cellular Morphology

(+) RNA viruses are dependent on cellular lipid metabolism and transport machinery for efficient genome replication. Phospholipid synthesis is significantly up-regulated [19,20,21] as early as 2 hpi in poliovirus infected cells [11]. Specific upregulated lipid species (Table 1) include phosphatidylcholine [19], sphingomyelin [11], and phosphatidylinositol-4-phosphate (PI4P) [22,23]. Fatty acid chain lengths of phosphatidylcholine are altered, with a longer acyl chain (C18/C18 and C18/C16) observed in infected cells as compared to mock-infected cells (C14/C16 and C16/C16) [24]. This alteration in the structural composition in poliovirus-infected cells indicates that membranes of poliovirus replication complexes are clearly distinct from pre-existing cellular membranes [25]. Lipid metabolism itself is critical for virus replication, as blocking de novo generation of phospholipids inhibits poliovirus RNA synthesis [26], indicating that poliovirus replication and virally induced lipid biosynthesis are coupled.

The mechanism by which the altered lipid metabolism induced by poliovirus infection contributes to virus replication is unclear. Various lengths of acyl chains and sizes of head groups of lipid components favor different membrane curvatures (reviewed in [27]). The spontaneous curvature caused by phosphatidylcholine (C10-14) is zero, forming flat lamellar structures [28]. Longer acyl chain lengths (C16-18) adapt phospholipids for forming more negative curvature membrane (formation of a concave surface) [29]. The longer acyl chains in infected cells [24] suggest that these newly synthesized phosphatidylcholines [11] may help induce the tubular deformation of proliferating membrane.

PI4P and cholesterol are enriched in the newly synthesized membrane structures and are critical for building poliovirus replication machinery and for efficient progeny RNA production. During infection, the phospholipid-modifying enzyme PI4KIIIβ is recruited to the replication site by poliovirus 3A (Table 1), resulting in a local elevation of its catalyzed product PI4P [23,30,31]. Since the poliovirus RNA-dependent RNA polymerase (3D) can bind directly to PI4P *in vitro*, a model has been proposed in which one of the functions of PI4P in poliovirus replication is to recruit and maintain association of soluble 3D within membranous replication factories [23,32]. This interaction could be mediated by the negatively charged head group of PI4P and the net positive charge of 3D. Additionally, PI4P incorporation has been demonstrated to increase membrane curvature by interacting with membrane-remodeling proteins via its head group [33,34] as has been shown in cells infected with hepatitis C virus (HCV) [35]. Such interactions are expected to induce membrane remodeling that facilitates efficient RNA replication.

**Table 1:** Poliovirus induced alterations in lipids and proteins.

Lipids/Proteins	Changes upon Infection (Superscripts Correspond to Methods Described in Next Column)	Experimental Methods
**Lipid Biosynthesis and Lipid Composition**		
Phosphatidylcholine	Synthesis increases ^a^ Fatty acid tail length increases (from C14/C16 to C18/C18) ^b^	Radioactive pulse-chase [11] Mass spectrometry [24]
Sphingomyelin	Synthesis increases ^a^	Radioactive pulse-chase [11]
PI4P	Synthesis increases ^a,b^	Radioactive pulse-chase [11] Protein lipid overlay assay [36]
Host long chain acyl-CoA synthetase	Activity increases ^a^ Long-chain acyl-CoA (phospholipid precursor) increases ^a^	*In vitro* measurement of newly synthesized fluorescent long chain acyl-CoA [24]
PI4KIIIβ	Generates PI4P ^a,b^	Fluorescence microscopy [23] Protein lipid overlay assay [36]
Poliovirus protein 2A	Activates free fatty acid import ^a,b^	Individual protein expression in cells [24] Measurement of uptake of fluorescent fatty acids [24]
Poliovirus protein 3A	Recruits PI4KIIIβ to replication machinery ^a,b^	Fluorescence Microscopy [23] Immunoprecipitation [23]
**Lipid Transport and Lipid Redistribution**		
PI4P	Recruits OSBP to replication machinery ^a,b^ May increase membrane fluidity ^c,d^	Immunoprecipitation [37] Fluorescent Microscopy [37] Fluorescence correlation spectroscopy [38] Fluorescence recovery after photobleaching [38]
Cholesterol	May counter over-fluidity caused by PI4P ^a,b^	Fluorescence correlation spectroscopy [39] Fluorescence recovery after photobleaching [38]
OSBP	Mediates PI4P/cholesterol exchange on the membrane ^a,b^	Chemical inhibitors: Itraconazole [37], OSBP ligands [40] *In vitro* liposome lipid-exchange assay [37]
Poliovirus protein 2BC	Activates PI4KIIIβ to generate more PI4P ^a,b^ Stimulates host OSBP activity ^a,b^	Individual protein expression in cells [40] Flow cytometry [40]
Poliovirus protein 3A	Recruits clathrin-mediated cholesterol-rich endosomes to replication machinery ^a,b^	Immunoprecipitation [41] Fluorescence Microscopy [41]

PI4P: phosphatidylinositol-4-phosphate, acyl-CoA: Acetyl coenzyme A; OSBP: Oxysterol-binding protein.

Cholesterol is a critical determinant of membrane fluidity and flexibility. It enters the cell from the extracellular medium through clathrin-mediated endocytosis from the plasma (reviewed in [42]), and is trafficked to replication factories with the aid of poliovirus protein 3A [41] (see Table 1). Formation of such cholesterol-enriched domains within replication membranes is proposed to counter potential over-fluidity and over-bending caused by the presence of the PI4P enrichment [41]. It has been reported that cholesterol helps build active virus replication complexes consisting of lipid rafts (detergent-resistant membrane microdomains that are enriched in cholesterol and sphingolipids), in HCV, where RNA and proteins are enclosed and protected from cellular immune responses [43,44]. Observed non-uniform distribution of free cholesterol, segregated into patches, in poliovirus-infected cells [41] strongly suggests lipid rafts play a similar role in poliovirus replication.

A question arose regarding how PI4P and cholesterol are transported and enriched at the site of virus replication. Oxysterol-binding protein (OSBP) has been demonstrated to be responsible for the transport of cholesterol and PI4P between the endoplasmic reticulum (ER) and Golgi [45], a pathway stimulated during poliovirus infection (see Table 1). Disruption of PI4KIIIβ or OSBP activity dramatically suppresses poliovirus RNA replication, possibly by obstructing the formation of viral replication complexes [40]. Together, PI4P and cholesterol enrichment, and synthesis of phospholipids with longer acyl chains, cooperate with viral proteins (discussed below) to modulate membrane remodeling for efficient RNA replication.

Membrane-associated poliovirus proteins are key participants in the membrane remodeling that is essential for viral RNA replication [46]. The role of individual proteins has been explored through gene expression or direct delivery of poliovirus proteins in cultured cells, with visualization of their cellular effects by electron microscopy. Results of these experiments are described in Table 2.

**Table 2:** Membrane remodeling induced by individual poliovirus proteins in the cell. Roles of individual poliovirus proteins in membrane remodeling.

Poliovirus Protein	Membrane Structures Induced upon Protein Expression/Delivery in Cells
2BC	Vesicles 50–350 nm in diameter [47,48] Single-membrane empty vacuoles, located in peripheral regions of the cells [49]
2C	Vesicles 50–350 nm in diameter [47,50] Clusters of large, clear single-membrane vesicles [50] Membrane structures of Myelin-like swirls (sectioned cross-sectionally) [47] Tubular sheets (visible when sectioned longitudinally) [47]
3AB	Upon direct delivery, dilates endoplasmic reticulum (ER) and Golgi lumen [51] Enlarged and aggregated vacuoles [51] Horseshoe structures [51]
3A	Swollen ER membrane [48,49] Dilated single-membraned tubular-structures [48,49]
2BC and 3A	Small double-membrane clustered vesicles [49] Large clear vacuoles [49]
2C and 3A	Swollen ER membranes enriched in cellular material with similar electron density to cytoplasm [49]

Based on the lipid biochemical experiments discussed above and the studies shown in Table 2, ER membrane structures have been proposed to be, at least partially, the origin of poliovirus-induced vesicles [30,49]. It has also been shown that an individual viral protein is capable of invaginating liposomes [51], suggesting that viral proteins directly, in the absence of cellular proteins, are sufficient for membrane remodeling. Optimal poliovirus replication is dependent on newly synthesized lipids (Table 1) and virus proteins (Table 2) that together produce efficient replication factories.

## 3. Viral Replication Cycle as Observed by Electron Microscopy

### 3.1. Overview of Cellular Changes

Poliovirus-infected cells display distinct morphological changes throughout infection. In overview, during early infection light microscopy data show a loss of chromatin from the central region of the nucleus and chromatin appears to be condensed near the nuclear membrane [17]. At 2.5 hpi, poliovirus-induced blebbing from the ER membrane is observed by electron microscopy [52], and at 3–3.5 hpi vesicles appear within the cytoplasm [52,53,54,55]. Large numbers of cytoplasmic bodies, originally called “U bodies”, were first observed by electron microscopy at 4–7 hpi [18]. Vesicles become more numerous over the course of infection, until the cytoplasm is vesicle-filled, as seen by electron microscopy of thin-sectioned cells [18,53,54]. Virus-induced vesicles are generally smooth-walled, with sizes that range from 50-200 nm in diameter. Late in infection, 7.5–9 hpi, cells round up and the cytoplasm has small and large clear vacuoles [18]. In addition, the nucleus is further distorted and can be difficult to distinguish, mitochondria are swollen, and U bodies are no longer visible. No further changes are observed in the cellular ultrastructure after this time and before cell lysis [56], and it has been suggested that major alterations in cell morphology beyond 7 hpi are an indication of the disruption of all physiological systems in the cell rather than related to virus replication [18].

**Figure 1:**
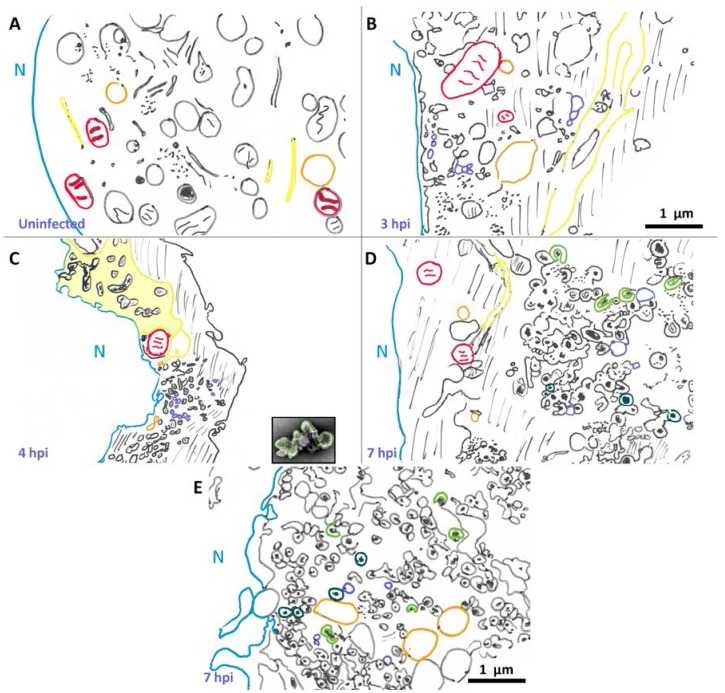
Changes in cellular morphology after poliovirus infection. Schematized cells from electron micrographs in Schlegel *et al.*, (**A**,**D**) [54], Dales *et al.*, (**B**,**E**) [53], and Mattern and Daniels (**C**) [57]. Representative structures are marked: nuclear membrane (blue), mitochondria (red), vacuoles (orange). Lines denote cytoplasm, and dots represent “viroplasm”. Endoplasmic reticulum (yellow) becomes enlarged and sometimes called “nuclear extrusions” (as in panel **C**). Early in infection single membrane vesicles (purple) are visible, and later the appearance of U bodies/horseshoe-shaped vesicles (green) and double membrane vesicles (teal). In panel **C**, an image of a fractionated rosette (from Figure 3) is shown to scale, highlighting its similarity to structures seen within infected cells. Scale bar 1 μm.

The morphology of poliovirus-induced vesicles has been investigated for nearly 60 years. Here we interpret these data, and discuss morphology information from these studies. While we are not able to include the original images, scaled, schematized versions are shown in Figure 1. It is important to note that while these drawings are our interpretation of the data, results from different groups show similar features and are consistent with what we describe here. A schematic of an uninfected HeLa cell, shown in Figure 1A, schematized from Schlegel *et al.*, 1996 [54], provides a point of reference for cell morphology changes that are visible in poliovirus-infected cells.

### 3.2. Morphological Details of Virus-Induced Membranes throughout Infection

The first morphological changes within the infected cell are apparent in the ER at 2.5 hpi [52]. Dramatic cellular changes are observed at 3 hpi. In 1965, Dales *et al.* [53] showed the appearance of single membrane vesicular bodies in the perinuclear region of the cytoplasm in infected cells at 3 hpi, as seen in Figure 1B. More recently, Belov *et al.* [55] collected three-dimensional data and have shown that what appeared to be vesicles in two dimensions are, in fact, inter-connected branching tubular structures. In addition to the appearance of single membrane tubular structures, the endoplasmic reticulum itself has undergone a transformation by 3 hpi. The lumenal space in the endoplasmic reticulum is enlarged, forming structures described as “nuclear extrusions” by Mattern and Daniel [57], as depicted in Figure 1C. These extended, altered endoplasmic reticulum-derived structures appear adjacent to newly formed perinuclear membranous structures. It is tempting to envision that poliovirus-induced vesicles derive from such nuclear extrusions.

By 1996, Schlegel *et al.* [54] were able to achieve enhanced sample preservation using high pressure freezing and freeze substitution. Their data show similar structures to those seen in earlier studies, with additional details visible. Particularly notable are double membrane structures preserved by tannic acid. Vesicles observed by these researchers 5 hpi (Figure 1D) are substantially different than those seen at 3 hpi. While single membrane vesicles are still visible at 5 hpi, there are now multiple clusters of poliovirus-induced vesicles evident within the cytoplasm, varying in location from perinuclear to peripheral. Vesicles within these clusters appear to be predominantly round or oval, and frequently display double membrane morphology. We note that some of these vesicles show lumenal content with increased densities compared to cytoplasm.

Even more striking is that some of these double membrane morphologies are not completely closed, but instead maintain a horseshoe-like appearance with a gated lumen that is connected to the cytoplasm through a neck approximately 15 nm in diameter. These structures appear similar in shape and size to U bodies described earlier [18] and resemble those discussed above in work by Dales *et al.* [53]. The U bodies may represent a transition state from single to double membrane vesicles or *vice versa*, but this is not yet clear. As seen in Figure 1D, at 5 hpi vesicles range in size from 100–200 nm in diameter, and clusters of vesicles are on the order of 1–2 μm across.

By 7 hpi (Figure 1E, schematized from [53]), there are more double membrane vesicles, as well as fewer single membrane vesicles. The changes are not as dramatic from 5 to 7 hpi as they are from 3–5 hpi, but poliovirus capsids are visible dispersed through the vesicle clusters, occasionally appearing as crystals, and frequently within the lumen of double-membrane vesicles.

Electron tomography data from Belov *et al.*, 2012 [55] revealed that the three-dimensional morphology of poliovirus vesicles is even more complicated than what had been visualized in two dimensions. At 3 hpi, vesicles in the cytoplasm consist of irregularly shaped, branching clusters of single membrane vesicles. The diameters of individual vesicles range from approximately 25–300 nm. These tubular vesicles appear tightly clustered, with their morphologies influenced by neighboring vesicles. At 4 hpi, individual vesicles appear larger in volume and some have associated viruses on their cytoplasmic side. At 7 hpi, most of the vesicular structures are composed of double membrane vesicles, with tightly apposed membranes. These more oval structures appear to be better separated, and less convoluted, with larger interior regions than the membranous structures visible at 3 and 4 hpi. As mentioned above, some of these double membrane structures appear to have gated lumenal areas, with a roughly round central lumen of diameter ~250 nm and a gate opening of ~20 nm. These three-dimensional data may correspond to similar horseshoe shaped structures observed earlier [18,54].

## 4. Cell Fractionation for Investigation of Poliovirus Replication Factories

**Figure 2:**
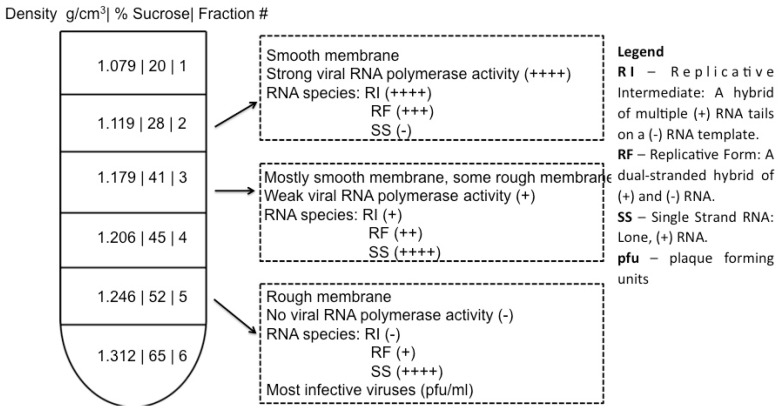
Subcellular fractionation of functionally distinct vesicles associated with replication processes. In work by Caliguiri and Tamm, Dounce homogenates of HeLa cells were layered in a discontinuous sucrose gradient, centrifuged at 86,000 x *g*. Upon fractionation of the gradient, membrane bands with the indicated densities were collected. Properties of fractions 2, 3, and 5 are shown. Vesicle content was examined by electron microscopy [58]; RNA polymerase activity of the isolated fraction was measured by tritiated ATP incorporation; the abundance of RNA species (RI, RF, SS) was determined by gel electrophoresis; and viral titer was measured by plaque assay [58,66]. Note: data from Caliguiri and Tamm [58,66,67].

Fractionated samples may represent isolation of clusters of poliovirus vesicles. Membrane-associated cellular and viral processes such as virus replication and protein translation are well separated by subcellular fractionation, as shown in Figure 2. The work of Caliguiri and Tamm [58] showed that sucrose density fractions arising from infected cells display significant increases in lipids and that the fraction containing twice the phospholipid content, as compared to the corresponding mock-infected sample, comprises vesicles with a smooth membrane (Fraction 2 in Figure 2) and contains newly synthesized phospholipids. The relative amount of smooth membrane increases during the course of virus infection, as compared to rough membrane [59]. Both Caliguiri and Mosser [60] and Butterworth *et al.* [61] provided evidence that the most abundant protein in this smooth membrane fraction is a 37 kDa polypeptide “X”, which has since been renamed poliovirus protein 2C (see [62] for a table of poliovirus protein nomenclature). Poliovirus protein 2C is associated with membrane remodeling (see Table 2), and is known to play a critical role in virus replication, which occurs on smooth membrane. A fraction containing rough membrane (membrane decorated with ribosomes; Fraction 5 in Figure 2), contains the majority of infectious virus. These data indicate that fractionation successfully separates RNA replication factories from virions as evidenced by a discrete distribution of replicative intermediates. This ability to resolve, by buoyancy, vesicles associated with infectious virus from those associated with RNA production, indicates that vesicles associated with replication are distinct from those associated with protein translation or virion packaging.

**Figure 3:**
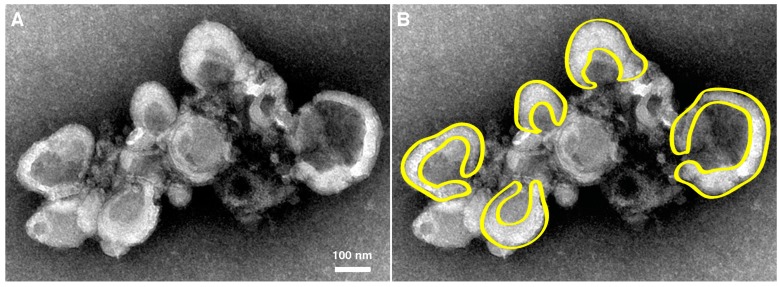
Morphology of rosette isolated from poliovirus-infected cells. (**A**) U body-like densities surround a dense central region of smaller vesicles. Cell fractionated material was prepared and imaged by Evan Rossignol using methods from Schlegel *et al.*, 1996 [54]. Sample from 4 hpi is negatively stained and imaged by electron microscopy; (**B**) Cartoon highlights the horseshoe-shaped (U body-like) configurations of individual vesicles of the cluster. Magnification bar, 100 nm.

Within Fraction 2 (Figure 2), replication complexes active in replication have been named “rosettes” and characterized by the work of Bienz *et al.*, 1990 [63]. As seen in Figure 3, rosettes appear as multivesicular aggregates, the overall configuration appearing as an oval shape approximately 1 μm by 500 nm, with an unknown height. These structures were called rosettes because they are composed of large (200–400 nm diameter) vesicles (“petals”) surrounding a more compact central structure [54,63,64,65]. While rosette structure is not necessary for RNA replication [21], the overall organization and morphology between isolated samples and clusters seen in sections prepared from virus-infected cells appears to be conserved, as seen in a to-scale schematic of Figure 3 included in panel D of Figure 1.

## 5. Conclusions

**Figure 4:**
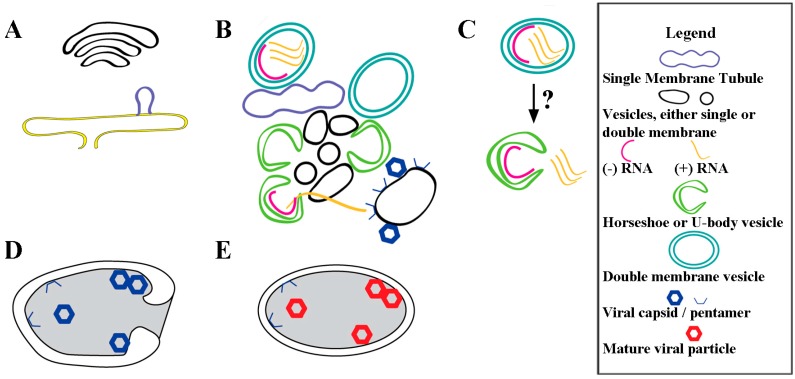
Virus-induced membrane remodeling and expansion for poliovirus replication. (**A**) As early as 2.5 hpi, lipid buds off the ER [52], producing structures comprised of mostly virally induced lipids [11]; (**B**) New and pre-existing cellular vesicles are dynamically remodeled [55] and invaginated from tubules into vesicles, and U-bodies, using a unique lipid composition and specific virus proteins (listed in Table 1 and Table 2). Only a fraction of the membranous structures are involved in active RNA replication within lumenal spaces. Throughout infection (+) RNA exits the replication factory, destined for translation or packaging. For virus assembly, (+) RNA interacts with membrane-associated capsid protein pentamers on membranes that are distinct from replication factory membranes [70]; (**C**) Possible mechanism by which closed DMVs transitions to U-body with gated lumen. These structures are visible at timepoints beginning 4 hpi and are believed to continue active replication; (**D**) Autophagosome-like vesicle formation engulfs immature virus particles. These vesicles have twice the diameter of replication vesicles [71], and we posit that these membranes are distinct from those in RNA replication factories, which are still present in the cell; (**E**) Acidification of the autophagosome-like vesicles produces mature virus particles [72].

During poliovirus infection there is a continuum of membranous structures that are formed in concert with assembly of replication factories and in support of RNA replication, as shown schematized in Figure 4. Virus-induced membranous structures begin to appear at 2.5 hpi [52] (Figure 4A), and dominate the cytoplasmic space by 7 hpi [18,53]. These structures serve multiple roles in virus-infected cells, including as a site for RNA replication [68] (Figure 4B,C), for RNA packaging (Figure 4B) [69,70], and for virus maturation [71,72,73,74,75] (Figure 4D,E). It is not clear whether membranous structures transition between roles or are independently formed. Indeed, if there are independent membrane formation paths for different viral roles, this could explain the observed presence in replication factories of markers from different cellular origins (ER, Golgi, *etc.*).

On a cellular scale, RNA synthesis is compartmentalized in infected cells. Negative-sense RNA is synthesized in a perinuclear region of ER, and (+) RNA in a more peripheral location [76]. Despite the fact that packaging of RNA is coupled to its replication [77], differing buoyant densities of fractionated vesicles indicate that packaging of RNA into virions occurs in a population of vesicles separate from those active in replication [64]. This suggests spatial compartmentalization of the viral processes, where (+) RNA migrates from the membranous site of RNA synthesis to membranous packaging sites, as shown in Figure 4B. Thus active replication involves only a fraction of the available membranous structures.

Early in infection poliovirus morphology and other (+) RNA viruses all appear to produce single membrane tubular structures [55,78]. The progression of the structures to double membrane vesicles is also shared. Evidence shows that RNA replication occurs within vesicle lumenal regions for multiple (+) RNA viruses (e.g., [79,80,81,82]). These structures may serve to sequester from host cell defenses the double-stranded RNA intermediate during RNA synthesis. In the case of a prototypical arterivirus, equine arteritis virus, the double membrane vesicles formed during infection label strongly for double-stranded RNA. These labeled vesicles display the same dark phenotype seen in a population of poliovirus vesicles. Analysis of the phosphorous content using electron spectroscopy reveals these vesicles in arterivirus infection contain the phosphorous content equivalent to roughly a dozen copies of the viral genome [81]. Although the method by which RNA products can exit the lumen of a double membrane vesicle is unclear for some viruses, poliovirus-infected cells display a possible intermediate. The U-bodies or horseshoe vesicles commonly observed late in poliovirus infection [18,53,54] display a gated lumenal morphology that is consistent with membrane fusion between the outer and inner vesicles, a feat possibly mediated in part by 3AB [51] (see Figure 4C).

Autophagosome-like vesicles are large double membrane structures, about twice the size of U bodies. They play a critical role in maturation of viruses, through acidification of the vesicle, which promotes the final step in virus maturation and viral transmission [46,71,72,83,84]. Better understanding of the pathways of vesicle transitions can help elucidate the infection cycle and the compartmentalization of function.

The complex cellular changes that occur throughout infection require specialized phospholipid biosynthesis that evolves over the time of infection [11,24,59]. In our model each vesicle population serves specialized roles (and perhaps some shared roles) that support the poliovirus replication processes. Different membranous species can co-exist temporally, providing a complex environment for efficient coordination of all aspects of poliovirus replication.
